# Computer-controlled liquid-nitrogen drizzling device for removing frost from cryopreserved crystals

**DOI:** 10.1107/S2053230X2001420X

**Published:** 2020-11-25

**Authors:** Yuki Nakamura, Seiki Baba, Nobuhiro Mizuno, Takaki Irie, Go Ueno, Kunio Hirata, Sho Ito, Kazuya Hasegawa, Masaki Yamamoto, Takashi Kumasaka

**Affiliations:** a Protein Crystal Analysis Division, Japan Synchrotron Radiation Research Institute, 1-1-1 Kouto, Sayo-cho, Sayo-gun, Hyogo 679-5198, Japan; b Advanced Photon Technology Division, RIKEN SPring-8 Center, 1-1-1 Kouto, Sayo-cho, Sayo-gun, Hyogo 679-5148, Japan; c ROD (Single Crystal Analysis) Group, Application Laboratories, Rigaku Corporation, 3-9-11 Matsubara-cho, Akishima-shi, Tokyo 196-8666, Japan

**Keywords:** protein crystallography, cryocrystallography, removal of frost, liquid-nitrogen drizzling device, automated data collection

## Abstract

Cryocrystallography is a technique that is used more often than room-temperature data collection in macromolecular crystallography. One of its advantages is the significant reduction in radiation damage, which is especially useful in synchrotron experiments. Another advantage is that cryopreservation provides simple storage of crystals and easy transportation to a synchrotron. However, this technique sometimes results in the undesirable adhesion of frost to mounted crystals. The frost produces noisy diffraction images and reduces the optical visibility of crystals, which is crucial for aligning the crystal position with the incident X-ray position. To resolve these issues, a computer-controlled device has been developed that drizzles liquid nitrogen over a crystal to remove frost. It was confirmed that the device works properly, reduces noise from ice rings in diffraction images and enables the centering of crystals with low visibility owing to frost adhesion.

## Abstract 2

Cryocrystallography, which is commonly used in macromolecular crystallography, may sometimes reduce the quality of diffraction data and the visibility of crystals owing to frost adhesion. A device has been developed to remove frost by drizzling liquid nitrogen over the crystals, which enabled noise reduction of diffraction images and the centering of crystals with low visibility owing to frost adhesion.

## 1. Introduction   

In macromolecular crystallography (MX) using a synchrotron, cryocrystallography is commonly used to obtain high-quality diffraction data (Hope, 1988 ▸; Parkin & Hope, 1998 ▸; Rodgers, 1994 ▸; Garman & Schneider, 1997 ▸). The sample temperature is maintained at 100 K or below using a cryostream device and is important for reducing radiation damage to the samples and automating sample exchange (Murakami *et al.*, 2012 ▸).

However, a major obstacle in this technique is ice or frost contamination in or on the flash-cooled droplets containing crystals. This deteriorates the diffraction images by the addition of reflections derived from ice diffraction. Frost adhesion and devitrification of solvent ice also lower the optical visibility of crystals, thus interfering with the process of aligning the crystal position with the incident X-ray position.

Ice formation is basically resolved by optimizing the harvesting solvent and/or cooling rate during the cryo­preservation process (Harrison *et al.*, 2019 ▸; Warkentin *et al.*, 2006 ▸), but the problem of frost mainly comes from the sample-handling environment. High humidity causes the condensation of water vapor contained in the air on cooled materials, such as samples, the sample changer, goniometer head and cryocooler nozzle. Even though this can be avoided by heating the devices (Russi *et al.*, 2016 ▸) or using dry air such as a sheath gas stream in a cryostream, it is inevitable in some cases, *i.e.* during transportation of the samples using dry shippers. Such frost is formed in the atmosphere and then adheres to the sample; therefore, frost removal is possible in many cases.

There are methods for removing frost from crystals mounted on goniometers. One is annealing (Harp *et al.*, 1998 ▸; Yeh & Hol, 1998 ▸), which is mostly used to improve crystal quality by removing the crystal disorder that is introduced during fast quenching (Kriminski *et al.*, 2002 ▸). To increase the temperature of crystals, blocking of the cold stream is suitable for automatic operation (Yeh & Hol, 1998 ▸) and is applied at the SPring-8 MX beamlines. This defrosting method can effectively remove frost, but some fragile samples may be damaged by the temperature change. There is also a method that involves rinsing the sample pins in the liquid nitrogen (LN2) dewar with a sample-handling robot; however, there is the possibility of frost adhering again (Soltis *et al.*, 2008 ▸). Another method involves flashing a refrigerant medium such as liquid nitrogen over the crystal (Pflugrath, 2004 ▸; Warkentin & Thorne, 2007 ▸; Garman & Owen, 2006 ▸). This method can remove frost and dust from a crystal by liquid flow. Even though some of the fixed dust still remains with certain flow rates and amounts of liquid, a sample can maintain a low temperature during the process.

The flashing-of-refrigerant method is advantageous for any sample. However, there are issues with its implementation on synchrotron beamlines. Recent MX beamlines are highly automated and many probes and instruments are densely packed around the sample position. In this situation, LN2-flashing devices must be compact and prevent the spillage of LN2 onto the other devices. In addition, computer-controlled operation is required because automation and remote measurements are widely carried out (Ueno *et al.*, 2006 ▸, 2016 ▸; Hirata *et al.*, 2019 ▸). Thus, the automatic sequence of setting up data collection must be carried out without human intervention and users must be able to apply the LN2 to the sample when they are controlling the experiment remotely. To solve these problems, we developed a computer-controlled device that automatically drizzles LN2. We describe its configuration and the results of diffraction experiments with and without the device.

## 2. Device and usage   

### 2.1. Device configuration   

**Figure 1:**
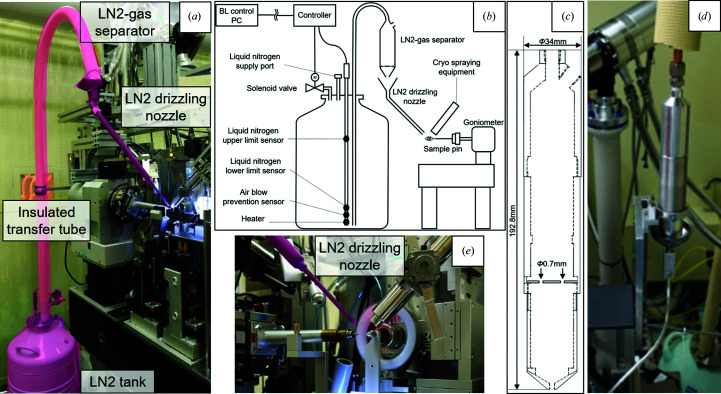
(*a*) The device at BL41XU. (*b*) A configuration diagram of the device. (*c*) A drawing of the LN2–gas separator. (*d*) The LN2–gas separator at BL26B2 (before installing the insulation tube). (*e*) A close-up view of the device at the sample position (the device is shown in pink).

The device consists of four major components, as shown in Fig. 1 ▸(*a*). The dewar tank is used to store LN2 and is equipped with a heater unit and a solenoid valve. To blow out LN2, the heater is turned on and the valve is closed by the increasing gas pressure inside the dewar (Fig. 1 ▸ *b*). The dewar also has upper- and lower-limit fluid-level sensors that are used when refilling the LN2. The LN2 is blown out through an insulated transfer tube and is transferred to an LN2–gas separator. The blowout contains nitrogen gas, which causes intermittent LN2 flow. Therefore, the nitrogen gas should be discarded using the separator, which is made of a cylindrical aluminium tube with a filter plate with four 0.7 mm diameter holes (Fig. 1 ▸ *c*). Only LN2 flows through these holes and passes down to the next LN2 drizzling nozzle, and the gas flowing out from the LN2 passes through the top hole of the separator tube (Fig. 1 ▸ *d*). The gas is then completely removed by the funnel-shaped top of the LN2-drizzling nozzle (5 mm internal diameter) and LN2 is delivered to the sample position. The distance from the tip of the LN2-drizzling nozzle to the sample position is 15 mm.

This device is designed to be compact for installation without interfering with the sample position, diffractometer and other beamline devices such as the cryostream nozzle, beam stopper and collimator (Fig. 1 ▸ *e*).

In the development of this device, it was important to avoid blowing nitrogen gas, which can cause a temperature increase at the sample position. As mentioned previously, only LN2 is able to drizzle onto the sample by completely relieving pressure using the two stages of the LN2–gas separator and LN2-drizzling nozzle. The LN2 supply parts (dewar tank, heater unit, solenoid valve and level sensors) were procured from Taiyo Nippon Sanso Corp., Tokyo, Japan. The LN2–gas separator and LN2-drizzling nozzle are both standard metalwork products and cost a total of approximately 1000 dollars.

### 2.2. Applications in various measurement situations   

The device can be operated via computers installed at the SPring-8 MX beamlines. For on-demand operation, both on-site and remote users can operate it through the control GUIs provided by the MX beamline-control software *BSS* (Ueno *et al.*, 2005 ▸) and the remote-measurement software *SP*8*Remote* (Ueno *et al.*, 2016 ▸). Both interfaces simply provide one-button operation.

The device has also been integrated into automatic measurement systems. *DeepCentering* is a novel system which performs automatic crystal recognition and centering by using deep-learning technology (Ito *et al.*, 2019 ▸). This system is designed to analyze clean sample photographs; however, frost adhesion to the sample loop interferes with the correct extraction of the outline of the crystals. For this reason, LN2 is drizzled in advance using the device before taking a crystal photograph. Its operation is incorporated into the automatic centering protocol of this system.

The *ZOO* system for automatic diffraction measurements (Hirata *et al.*, 2013 ▸) also supports the device. In this system, crystal centering is performed using both optical image recognition and X-ray scanning. For image recognition, the loop is centered and oriented using the following steps: (i) the outline shape of the loop is recognized from the sample photograph and the center of the loop is adjusted to the X-ray position and (ii) the goniometer φ angle is determined to orthogonalize the loop plane against the X-ray beam axis by maximizing the projected area of the loop (facing). Facing is especially important for small wedge measurements, in which each microcrystal in the area is serially measured over a small φ-angle range. In the case of adhered frost, the outer shape of the nylon loop will not be detected accurately. Additionally, low accuracy in facing, *i.e.* the inclination of the plane of the loop, increases the path length of the X-rays through the sample, thus increasing the rate of multi-hit diffraction images, which are not suitable for data processing. Additionally, adhered frost results in noisy ice diffraction during data-set collection, which interferes with accurate measurements.

## 3. Results and discussion   

### 3.1. Device operation and visual inspection of crystals before and after drizzling LN2   

**Figure 2:**
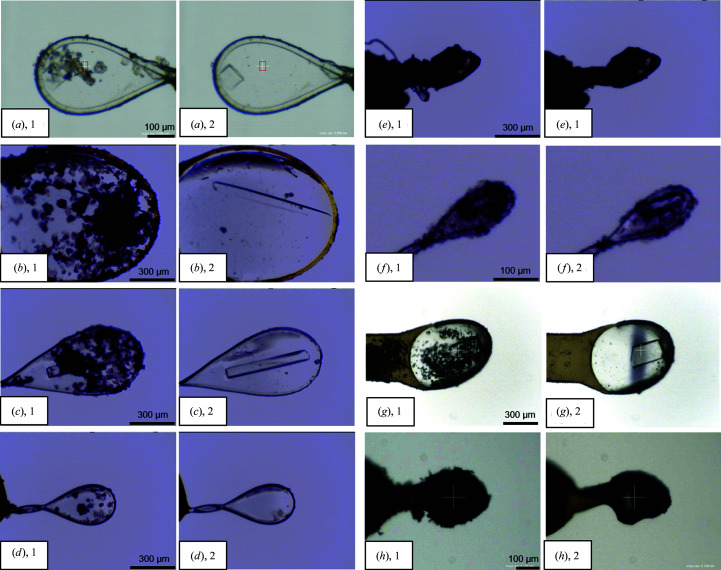
Examples of frost removal from samples. (1) Before and (2) after LN2 drizzling. Samples (*a*–*f*) are crystals from beamline users. In sample (*e*) fibrous dust was removed and in sample (*f*) very fine frost was not completely removed. Sample (*g*) is a lysozyme crystal and image (*g*), 2 shows a dark shadow caused by X-ray exposure. Sample (*h*) is the lysozyme crystal described in Section 3.3.

The device can continuously transport an LN2 flow to the sample position at a flow rate of 0.4–0.5 ml s^−1^. The rate varies depending on the amount of LN2 remaining in the tank, but it does not severely change the ability to remove frost. In our experience, one-time drizzling for about 10 s is sufficient to clean the crystals. This device can remove ice and fibrous dust adhering to the surface. However, it sometimes failed to completely remove very fine frost (Fig. 2 ▸ *f*). This type of frost, powder-like frost with dimensions of approximately a few micrometres, often occurs during long-term storage in dry shippers and might also occur at low temperatures and lower supersaturation conditions according to the Nakaya snow-crystal morphology diagram (Nakaya, 1954 ▸). In such a case, longer drizzling can be applied. However, the nozzle sometimes blocks up owing to the formation of frost on it when LN2 is drizzled continuously for more than 180 s.

Examples of sample-crystal photographs are shown in Fig. 2 ▸ and were taken using sample-observation CCD cameras installed on the beamlines. After drizzling, the adhered frost was completely removed and the crystal visibility improved. We confirmed that not only frost but also unidentified ice or dust was removed. The temperature variation of the samples owing to the drizzling was measured using a simple method involving a type-K thermocouple; the temperature dropped to 86 K from 127 K within 0.25 s after LN2 was drizzled.

### 3.2. Time of frost removal   

We compared the times required for frost removal by drizzling LN2 using the device and by using a manual LN2-drizzling tool (Pflugrath, 2004 ▸; Warkentin & Thorne, 2007 ▸). The experimental protocol was assumed to be a standard experiment at a SPring-8 MX beamline; that is, it includes automated sample exchange using a SPACE robot (Murakami *et al.*, 2012 ▸). The time was measured from immediately after setting the sample pin on the goniometer to starting data collection. In both cases, LN2 was drizzled for 10 s. The average time for each of the five samples was 106.0 ± 4.5 s for manual operation and 66.8 ± 5.8 s for the device. This shows that the device is about 40 s faster per sample. For manual operation, the time included an operator opening/closing the door of the experimental hutch to drizzle LN2, which took about 20 s.

### 3.3. Comparison of diffraction data before and after frost removal   

To evaluate the effect of frost adhesion in data collection, diffraction data were collected from the same crystal before and after frost removal using the device. The measurements were conducted under the same conditions as those reported for beamline BL26B2 at SPring-8 (Ueno *et al.*, 2006 ▸; Table 1 ▸). We used the tetragonal hen egg-white lysozyme crystal shown in Fig. 2 ▸(*h*).

The frost-adhered crystals were prepared using the following procedure: the crystals were picked up from a droplet with a cryoloop and held using cooled cryotongs (277 K). This was performed inside a cool workbench, which was operated at 277 K and located beside the beamline experimental hutch (Baba *et al.*, 2019 ▸). The crystal was transferred to the goniometer head and then flash-cooled at 100 K from the cryotongs. This cooling was carried out on the beamline at 308 K. The cooled cryotongs sometimes condense water vapor around the crystal during transportation.

**Figure 3:**
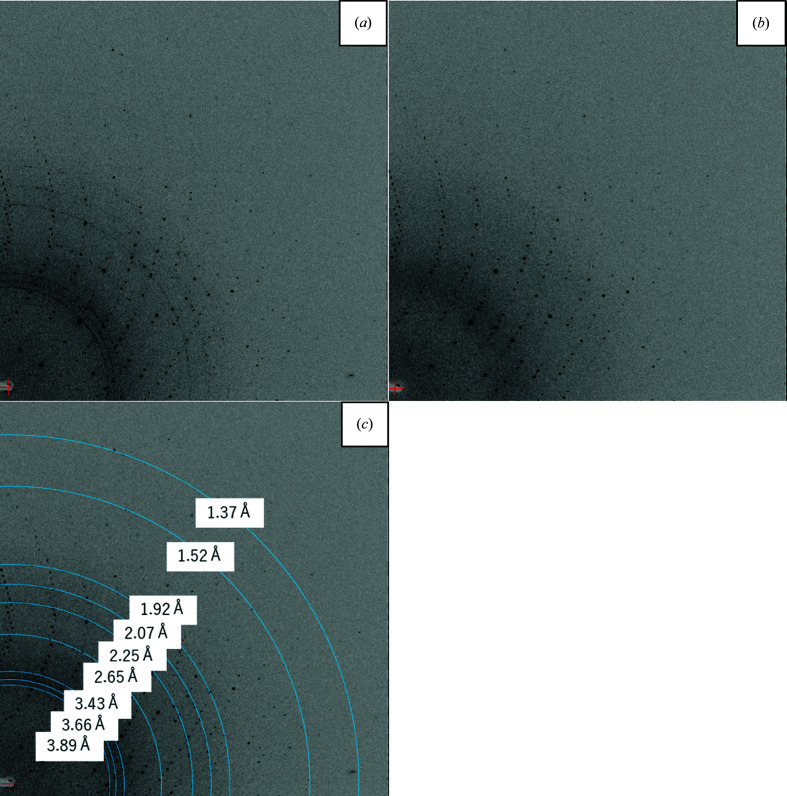
Diffraction images before (*a*) and after (*b*) frost removal. (*c*) The image in (*a*) with the Debye rings from ice crystals marked with the relevant resolutions (Malkin *et al.*, 2012 ▸; Chapman & Somasundaram, 2010 ▸). The ice rings and diffraction spots disappeared after drizzling the crystal with LN2.

The diffraction images were taken with the same oscillation range in both data sets, as shown in Fig. 3 ▸. Debye rings of ice diffraction were initially observed at all oscillation angles, but they completely disappeared after LN2 drizzling. Both rings correspond to ice-crystal reflections, as shown in Fig. 3 ▸(*c*).

**Figure 4:**
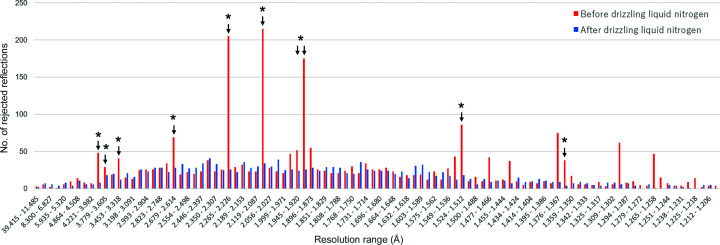
The number of rejected reflections in each resolution range. The difference in the applicable resolution ranges is extremely large. Asterisks indicate the resolutions marked in Fig. 3 ▸(*c*).

Each data set was processed using *XDS* (Kabsch, 1988 ▸), and the number of rejections in 100 resolution shells was determined, as shown in Fig. 4 ▸. In the shells relevant to ice diffraction, the statistics improved significantly. In addition, after removing the ice *R*_merge_ changed from 3.8% to 3.6% and *I*/σ(*I*) changed from 17.66 to 18.25 in the overall statistics (Table 2 ▸).

## 4. Conclusion   

The LN2-drizzling device worked well to clean the crystal samples. Use of the device to remove surface frost has the following advantages: (i) there is no diffraction from the attached frost and/or dust, improving the experimental data, and (ii) the visibility in sample photographs is improved. It is also suggested that the device can contribute to the success rate of crystal centering during automatic measurements.

Furthermore, the computer-controlled automation of LN2 drizzling (i) improves experimental throughput by shortening the operation time of drizzling and (ii) enables the implementation of LN2 drizzling in remote experiments.

We started the development of this device in 2016, and it is currently available on SPring-8 MX beamlines BL26B2, BL41XU (Hasegawa *et al.*, 2013 ▸) and BL45XU.

Even though some types of frost may not be completely removed, as stated above, the challenge for the future is to apply more pressure in the LN2 dewar to increase the flow rate of LN2. For this purpose, it will be necessary to ensure the safety of pressurization, to prevent dew condensation in the flow path and to prevent nitrogen gas from being blown out of the drizzling nozzle.

## Figures and Tables

**Table 1:** Measurement conditions

Beamline	BL26B2, SPring-8
Detector	Rayonix MX225HS
Wavelength (Å)	1.0000
Rotation range per image (°)	0.2
Total rotation range (°)	90.0
Exposure time per image (s)	0.2
Crystal-to-detector distance (mm)	110
Beam size (µm)	50 × 60
Beam shape	Gaussian
Photon flux (photons s^−1^)	1.52 × 10^10^
Maximum dose† (MGy)	0.425

† The absorbed dose was calculated using *RADDOSE*-3*D* (Zeldin *et al.*, 2013 ▸).

**Table 2:** Data-processing statistics before and after LN2 drizzling Values in parentheses are for the outermost shell.

	Before drizzling LN2	After drizzling LN2
Space group	*P*4_3_2_1_2	*P*4_3_2_1_2
Unit-cell parameters (Å)	*a* = 78.83, *c* = 36.95	*a* = 78.83, *c* = 36.95
Resolution range (Å)	39.41–1.20 (1.27–1.20)	39.41–1.20 (1.27–1.20)
No. of reflections (observed)	239029 (22035)	240059 (22199)
No. of reflections (unique)	66958 (9985)	67041 (10036)
Completeness (%)	96.2 (88.6)	96.4 (89.1)
*R*_merge_ † (%)	3.8 (75.6)	3.6 (75.8)
CC_1/2_ (%)	100.0 (51.0)	100.0 (51.7)
〈*I*/σ(*I*)〉	17.66 (1.18)	18.25 (1.16)

† *R*_merge_ = 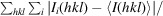 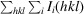.
